# Percutaneous mitral valve repair in patients developing severe mitral regurgitation early after an acute myocardial infarction: A review

**DOI:** 10.3389/fcvm.2022.987122

**Published:** 2022-09-23

**Authors:** Rodrigo Estévez-Loureiro, Marta Tavares Da Silva, José Antonio Baz-Alonso, Berenice Caneiro-Queija, Manuel Barreiro-Pérez, Francisco Calvo-Iglesias, Rocio González-Ferreiro, Luis Puga, Miguel Piñón, Andrés Íñiguez-Romo

**Affiliations:** ^1^Cardiovascular Research Group, Department of Cardiology, University Hospital Alvaro Cunqueiro, Galicia Sur Health Research Institute (IIS Galicia Sur), Servizo Galego de Saude, University of Vigo, Vigo, Spain; ^2^Department of Cardiovascular Surgery, University Hospital Alvaro Cunqueiro, Vigo, Spain

**Keywords:** mitral regurgitation, myocardial infarction, transcatheter mitral valve (MV) repair, cardiogenic shock (CS), MitraClip^®^

## Abstract

Acute mitral regurgitation (MR) may develop in the setting of an acute myocardial infarction (AMI) because of papillary muscle dysfunction or rupture. Severe acute MR in this scenario is a life-threatening complication associated with hemodynamic instability and pulmonary edema, and has been linked to a worse prognosis even after reperfusion. Patients treated solely with medical therapy have the highest mortality rates. Surgery has been the only treatment strategy until recently, but the results of the technique are hindered by high rates of morbidity and mortality. Therefore, the development of less invasive interventions for correcting MR would be ideal. We aimed to review the current role of transcatheter interventions in this clinical setting.

## Introduction

Acute mitral regurgitation (MR) may develop in the setting of an acute myocardial infarction (AMI) because of papillary muscle dysfunction or subvalvular apparatus rupture. This is a high-risk complication with a prevalence up to 3% of AMI patients. This condition is more common in patients presenting with hemodynamic instability or pulmonary edema, and is associated with an impaired prognosis even in the era of primary angioplasty (1–4). Anatomically, there are various types of lesions that may end up in the development of MR. Complete papillary muscle rupture is uncommon but is often fatal without rapid correction, leading the patient to cardiogenic shock (5). Acute MR without complete papillary muscular rupture may induce as well severe MR due to the combination of leaflet tethering and left ventricular dilation produced by an adverse remodeling pattern or partial papillary muscle rupture and can lead to recurrent heart failure or cardiogenic shock (CS) during early after the event (6). The prevalence of the latter that may account for 35–55% of the cases, which means that severe MR without complete rupture is not so uncommon and may deteriorate patients' condition enough to require an intervention (4, 7). Surgery has been the standard of care until recently, but is associated with high rates of morbidity and mortality (up to 20–25%, in-hospital). Additionally, patients under isolated medical management present a dismal prognosis (8, 9). Therefore, the development of less invasive interventions for correcting MR in this scenario are appealing.

## Scope of the problem: Prevalence and prognostic impact

In the setting of AMI, MR is a common finding, but the prevalence varies in different studies. Early angiographic studies report a prevalence of 12–19% with ventriculography performed within the first 16 days after the MI (10–12). In echocardiographic studies MR was observed in up to 50% of patients with AMI (4, 13–16). However, these prevalence studies have some limitations that must be pointed out. While some registries were performed in the era of fibrinolysis, in others PCI was the treatment of choice. Also, MR could be assessed in the first hours of the acute MI or echocardiography performed even 16 days after MI. Moreover, MR could be previously present and not be related to the acute coronary syndrome. Nevertheless, the prevalence reported is higher than the prevalence of MR in general population and therefore it can be assumed MR as a common complication of acute MI. The proportion of different degrees of ischemic MR is similar in patients with and without ST-segment elevation myocardial infarction submitted to PCI (4, 13). In the majority of studies, mild MR is by far more frequent and in a recent retrospective single-center study which included a thousand patients with AMI, mild MR was more frequent (76%), followed by moderate MR (21%) and severe (in 3% of patients) (13). Those elderly patients, female patients and with clinical evidence of heart failure present more frequently with greater MR grades (17–19). More severe MR also correlates with the presence of multivessel disease and lower left ventricular ejection fraction (LVEF) (11, 16, 19).

As previously mentioned, there are two mechanisms that may lead to development of MR early after MI. Papillary muscle rupture (partial or complete) is a life-threatening complication of AMI with a prevalence estimated of 1–3% (20, 21). This mechanical complication causes acute severe MR with acute volume overload, pulmonary edema and cardiogenic shock, with in-hospital mortality rate up to 80% in patients managed conservatively (22–24).

But MR may develop as well because of LV dilation and remodeling and leaflet tethering, resulting in an acute/subacute form of functional ischemic MR. This entity has also an impact on prognosis. Any degree of MR is independently associated with mortality in patients undergoing PCI for acute MI with a relationship between the MR severity and outcomes (3, 11). There is a correlation between ischemic MR severity and myocardial viability, with viable myocardial reducing LV remodeling and preventing development or worsening of MR. Importantly, early reperfusion with PCI in STEMI patients is associated with lower incidence of this type of ischemic MR (4, 25).

## Imaging techniques

The diagnostic workup for patients developing MR after MI requires firstly a high index of suspicion, and, therefore echocardiography is paramount in the differentiation of the mechanism for the MR and excluding other causes for a new systolic murmur in patients developing heart failure postMI. In the acute MR setting left atrium is usually of normal size, and the sudden increase in left atrial pressure is transferred backwards into the pulmonary veins, causing a rapid developing pulmonary edema. This event may result as well in a poor transthoracic imaging window, and, subsequently requiring transesophageal echocardiography to confirm the presence and severity of MR.

Echocardiographic assessment should include careful assessment of LV (ejection fraction, dimensions and wall motion abnormalities), mitral valve structure (annulus, leaflets, chordae and papillary muscles), and quantitation of the degree of MR. An integrative approach to the evaluation of MR should be performed including qualitative, semi-quantitative and quantitative parameters according to imaging guidelines (26). Overall MR severity assessment, integrates LV size and function, left atrial size, impact on Doppler flows and predicted systolic pulmonary artery pressure. MR may also be a dynamic entity related to the occurrence of myocardial ischemia and may diminish or even disappear after it is corrected by PCI, so a re-assessment should be advisable after the revascularization.

Acute MR due to LV remodeling is a consequence of the loss in the normal spatial relationship between LV and the mitral valve complex. With adverse LV remodeling (dilatation and shape modification), one or both mitral leaflets are apically displaced into the LV and away from the center of the cavity due to the outward displacement of the papillary muscles. This pattern is best seen in the apical 3 and 4 chamber views. In this sub-entity, the leaflets are essentially normal and the mitral annulus may be dilated (primarily septal-lateral and to a lesser degree inter-commissural), although this is more frequent in non-acute MR setting. MR can develop both due to global or regional remodeling, but the specific remodeling site might be of relevance since inferior MIs are more likely to be associated with significant MR compared to anterior MIs. This is probably related to different tethering patterns. Most of the patients with symmetric tethering have central jets, whereas patients with asymmetric tethering have posteriorly directed jets.

**Figure 1:**
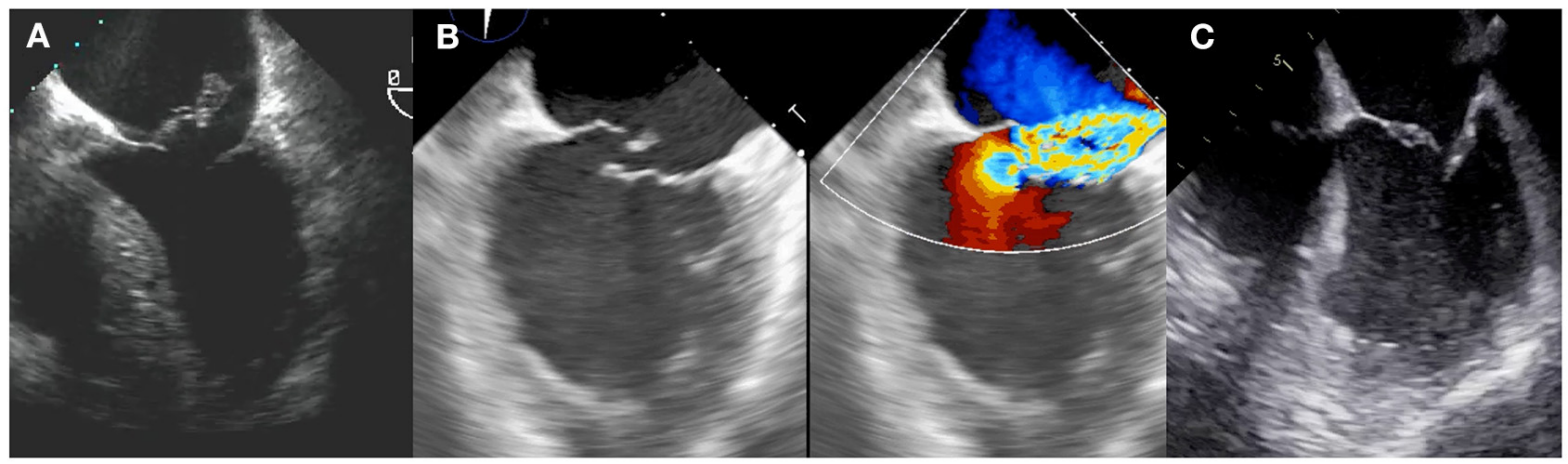
Types of postMI MR. **(A)** Complete papillary muscle rupture, with the papillary head flailing into left atrium. **(B)** Partial papillary muscle rupture. **(C)** Functional mechanism.

The most severe form of acute MR is papillary muscle rupture. Common two-dimensional echocardiographic features include a mitral leaflet flailing into left atrium together with severed chordae or a papillary muscle head bouncing within the left heart chambers. Complete avulsion of the papillary muscle is quite unfrequent in the primary PCI era, whereas a partial rupture or a tip rupture are more common. Posteromedial papillary muscle is more commonly affected than the anterolateral and this is related to the blood supply pattern. LV is frequently supranormal because of an abrupt decrease in afterload, and wall motion abnormalities can be undetected. Underestimation of the degree of MR by color Doppler is common due to the eccentricity of the jet. A summary of the types of MR after MI are shown in Figure 1.

In an acute setting, other imaging techniques such as cardiac computed tomography or cardiac magnetic resonance are less commonly performed. In a non-acute scenario, LV fibrosis location and extension, assessed by late gadolinium enhancement in cardiac magnetic resonance, have been related reverse remodeling or clinical outcomes in patients undergoing surgical or transcatheter mitral correction (27, 28).

## Surgical treatment

Surgery has been the standard approach and the only option for patients who develop MR early after MI and who remain symptomatic despite revascularization until last years. The optimal surgical approach to this entity must take into consideration the mechanism underlying regurgitation. Papillary muscle rupture or ruptured chordae, causing severe acute MR is a very poorly tolerated condition, where prompt mitral valve surgery could be lifesaving. Even though urgent surgery with mechanical assistance after MI is supported due to the risk of abrupt decompensation, deferring intervention provides time for the development of fibrotic tissue and is associated with lower surgical mortality, especially in patients without initial hemodynamic instability neither fulfill criteria for shock (29, 30). One study identified a median time to surgery of seven days (7). Ultimately in patients with cardiogenic shock emergent surgery is linked to increased survival when is promptly performed (29). The 2020 ACC/AHA Guidelines for Valvular Heart Disease, recommend, if possible, mitral valve repair, especially if papillary muscle rupture is not complete and the tissue quality is suitable for repair (31). However, mitral valve replacement is more commonly performed because of greater reproducibility, and established durability in patients with a high adverse event rate (32). Surgical revascularization at the time of valve intervention does not seem to influence the acute postoperative course (33).

Although surgical interventions are associated with better outcomes than conservative management (8, 9), the results of the technique are blunted by a still high early mortality due to the performance of a significant aggressive procedure in patients with poor clinical condition and an ongoing ischemic/scarred myocardium. In a recent review of surgical series in acute MR, 8 series of cases reporting results on surgery vs. conservative management were analyzed (8). Overall early surgical mortality was 19.2%. Of course, is lower than the 51.4% reported in the medical arm, but it is still very high, taking into consideration that those patients included in these retrospective registries (since there is no randomized trial in this setting) are those who were selected to be operated and therefore, probably biased to have a better survival chance. Taking these facts into consideration, the development of new less aggressive interventional techniques to correct acute MR is really appealing.

## Role of transcatheter interventions

The transcatheter options for MR treatment have grown exponentially during the last years. From all devices available, the edge-to-edge technique with the MitraClip system (Abbot Vascular, Santa Clara, USA) represents by far the most used and accumulates the larger clinical experience. The edge-to-edge repair (TEER) with MitraClip has been shown to be an efficacious device for correcting MR and it has been linked to clinical improvement both in primary and secondary MR (34–38). However, most MR cases are performed on patients in chronic and stable clinical situation, and, therefore, patients with acute MR are barely included in registries or randomized trials. Therefore, since acute MR represents a large unmet need in the development of less invasive treatments, the experience with TEER in this scenario has grown significantly in the last years.

**Figure 2:**
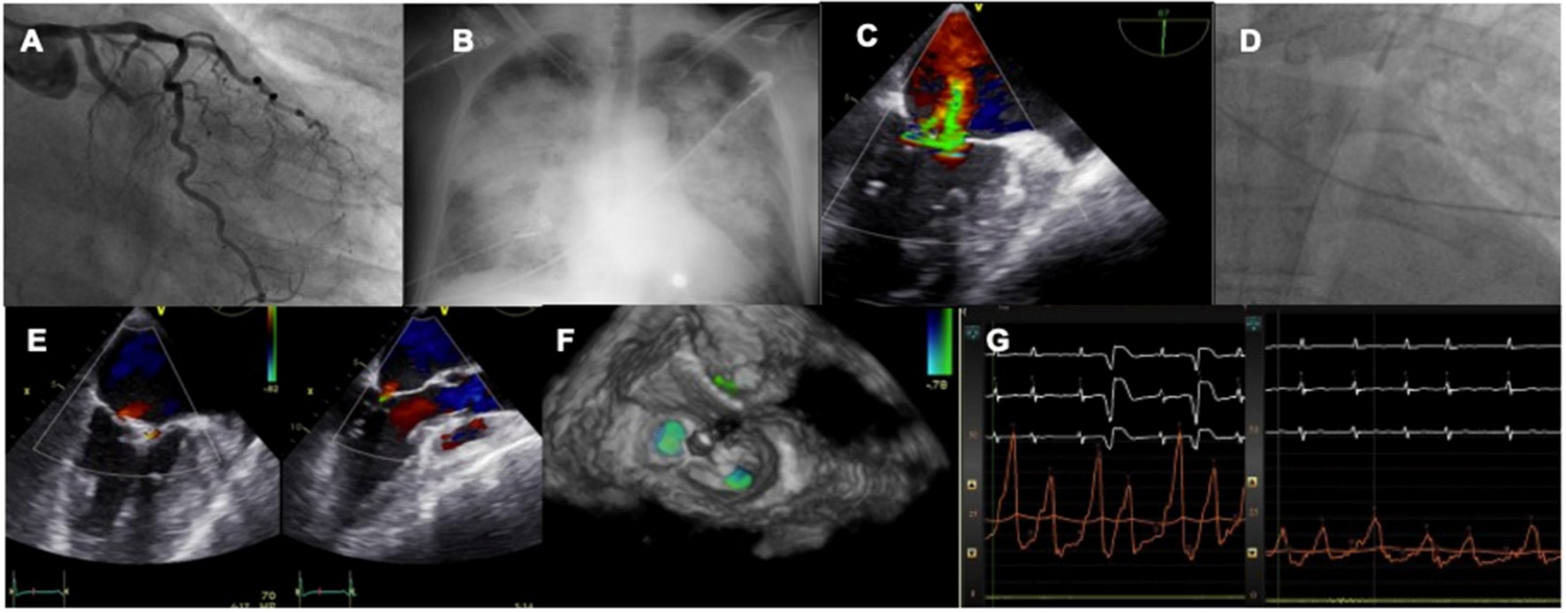
Case of acute MR after MI treated by TEER. A patient with LCX myocardial infarction **(A)** develops rapid pulmonary edema **(B)** and severe MR is diagnosed with echo **(C)**. An IABP is inserted to stabilize the clinical condition **(D)**. The valve is repaired with two MitraClip **(E,F)** leading to an acute drop in left atrial pressures **(G)**.

The first experiences with MitraClip were case reports and small series of cases showing the feasibility of treating this complex scenario with a percutaneous device in both cases of subvalvular apparatus rupture or those more functional (39–44). In that series of extreme risk patients TEER was associated with significant clinical and hemodynamic improvement, setting the field for larger registries to come (Figure 2).

Subsequently, the IREMMI group published the larger series on the topic. The first paper was published in 2020 showing the European experience with MitraClip in this setting (45). Forty-four patients with a mean age of 70 ± 10.8 years were included between 2016 and 2018. Interestingly, median time between MI diagnosis and treatment was 18 days and between development of MR and treatment 12.5 days. Patients were highly symptomatic with 63.6% in NYHA IV at the moment of the procedure and 68.2% received acute mechanical reperfusion due to MI. Median EuroScore II was 15.1%, thus representing the high-risk of the cohort and 16 patients received mechanical cardiac support (14 intraortic balloon pump, IABP, and 2 VA ECMO). In this series technical success was 86.6%. During follow-up, mortality at 30 days was 9.1%, representing a more than acceptable figure for such a high-risk cohort without surgical options. At 6-month MR ≤ 2+ was noted in 72.5% and NYHA I–II was observed in 75.9% of surviving patients.

**Figure 3:**
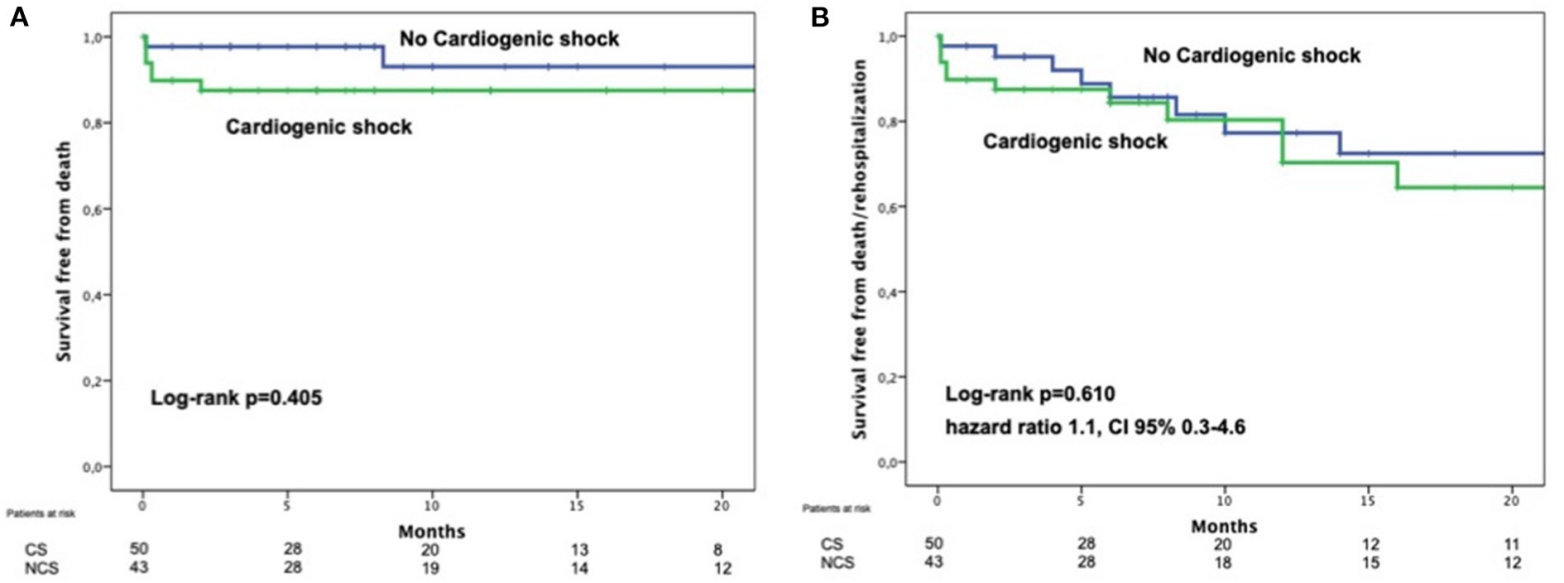
Comparison of survival free from death **(A)** or death and heart failure **(B)** of patients with postMI MR treated by TEER comparing those on cardiogenic shock with those who were not in cardiogenic shock. With permission from Haberman et al. (44).

In the next registry authors investigated the role of cardiogenic shock (CS) in the outcomes of a cohort of 93 patients with TEER after acute MR due to MI (46). Ninety-three patients in this scenario were included in this investigation, mean age 70.3 ± 10.2 years, and with 53.8% deemed to be in CS at the time of MitraClip implantation. 66% of the patients in CS were under support with IABP/Impella and 12% under VA ECMO. Technical success was high and did not differ between groups. Interestingly, 30-day mortality, although higher in CS group, was not statistically significant between groups (10% CS vs. 2.3% non-CS; *p* = 0.212). It is relevant to point out that the mortality rate in those patients non in CS was extremely low, even for such a high-risk population. Likewise, the combined event mortality/re-hospitalization was comparable (28% CS vs. 25.6% non-CS; *p* = 0.793) and the MR reduction at 3-months was as well similar (Figure 3) after 7 months of follow-up.

Of interest, the only variable associated with clinical outcomes was the procedural success. Therefore, authors claimed that CS should not preclude a treatment with MitraClip in this group and the essential point is to have enough experience in the team to ensure an adequate result.

In another paper from the group, authors analyzed the effect of the left ventricular ejection fraction (LVEF) on outcomes of 105 patients receiving MitraClip for MR early after MI (47). Authors divided the cohort in a LVEF cut-off of 35%. Up to 1 year, mortality rates were comparable between groups (11 vs. 7%, *p* = 0.51 and 19 vs. 12%, *p* = 0.49) and neither was re-hospitalization rate at 3-month follow-up. Therefore, the positive effect of percutaneous treatment is sustained in those patients with lower ejection fractions.

**Figure 4:**
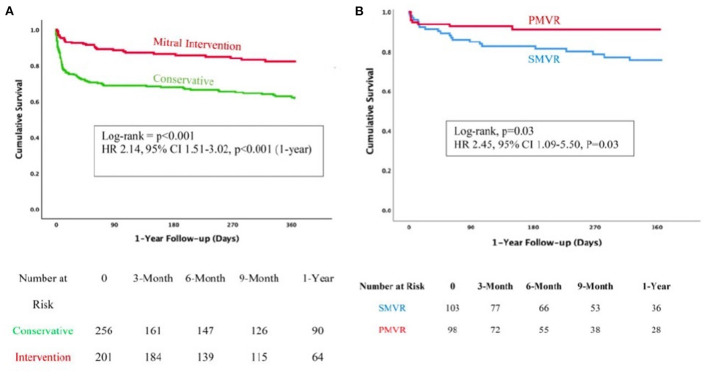
Comparison of patients with post MI MR under medical management vs. intervention **(A)** or surgery vs. TEER **(B)**. With permission from Haberman et al. (9).

Finally, the most comprehensive paper from the group compared three strategies of management of MR early after MI, conservative, surgical and TEER (9). A total of 471 patients were included in this registry (43% female, age 73 ± 11 years):266 were managed conservatively and 205 underwent interventions, of whom 106 were surgical management and 99 TEER. In line with previous surgical literature, those patients managed medically presented the worst outcomes with two-times more mortality than those who received an intervention. However, more interesting is the comparison of both interventional strategies. The article shows that those patients undergoing surgical correction presented worse outcomes than those receiving MitraClip, with a more than two-fold increase of mortality at 1 year. This difference was mainly driven by the mortality during hospitalization phase (16 vs. 6%, *p* = 0.03). And this finding was independent of the risk score profile of the patients (Figure 4).

Interestingly, this result was maintained even after propensity score adjustment and considering that patients in the TEER arm were older and had higher morbidity burden. However, only functional type MR was included in this investigation and therefore, results are limited to this subgroup.

Taking into consideration the positive results of this therapy in all the available literature we can conclude that there are several potential advantages on this treatment. First, the rapid hemodynamic improvement induced by the relief of MR with decrease in left chambers and pulmonary artery pressures and the increase of cardiac output, which may lead to a faster recovery (48). Second, the avoidance of the inflammatory reaction induced by the extracorporeal circulation necessary for surgical correction that can induce further LV damage (49). Moreover, MitraClip can avoid the restriction in the annular motion caused induced by prosthesis or surgical rings and the development of abnormal septal motion that can affect LV contractility and efficiency. In addition, this entity usually develops in a normal leaflet mitral valve, which usually present optimal leaflet tissue and anatomy for the device. Of relevance, TEER does not interfere with a delayed cardiac surgery in case the device fails or recurrent MR is present. And finally, TEER is associated with lower bleeding complications, a fact that can negatively affect an open-heart surgery, in patients usually at high bleeding risk due to the antithrombotic therapy related to post MI management.

However, we face some challenges and limitations as well when opting for this strategy. MitraClip in acute MR is a technically demanding procedure. Valve anatomy, a non-dilated atrium that complicates a precise transeptal puncture, the clinical status and the risk of entanglement in the subvalvular apparatus make these cases challenging. Nonetheless, and after taking these considerations into account, the procedure itself does not differentiate from the ones preformed in other clinical condition: increase coaptation surface or control flailing segments, decrease MR and obtain a drop in left atrium and pulmonary pressures.

Another question to consider is that procedures were performed in centers that had high levels of experience using MitraClip. Thus, TEER strategy cannot be generalized to less experienced teams. Likewise, clinical deterioration of some patients may be very fast, and this raises the question of whether specialized mitral teams should be prepared to deliver the therapy in emergent situations or whether they should even go to centers that do not offer it and whose patients are too unstable to be transferred.

Regarding limitations, several should be pointed out. Although results with TEER in this setting are promising, literature is limited to retrospective analysis of a small sample size. Therefore, we cannot exclude the presence of a selection bias in patients undergoing TEER, in the sense that only those who responded to the medical therapy and cardiac support were those who received the therapy. This population can represent a better prognostic category and therefore our conclusions may not be applicable to all patients. In addition, long-term clinical and echocardiographic follow-up is limited. Ideally, the implementation of a properly designed and executed randomized trial should provide more reliable information. However, as in surgical literature, this trial is still lacking. Further research must be warranted to elucidate the best management options for this condition.

**Figure 5:**
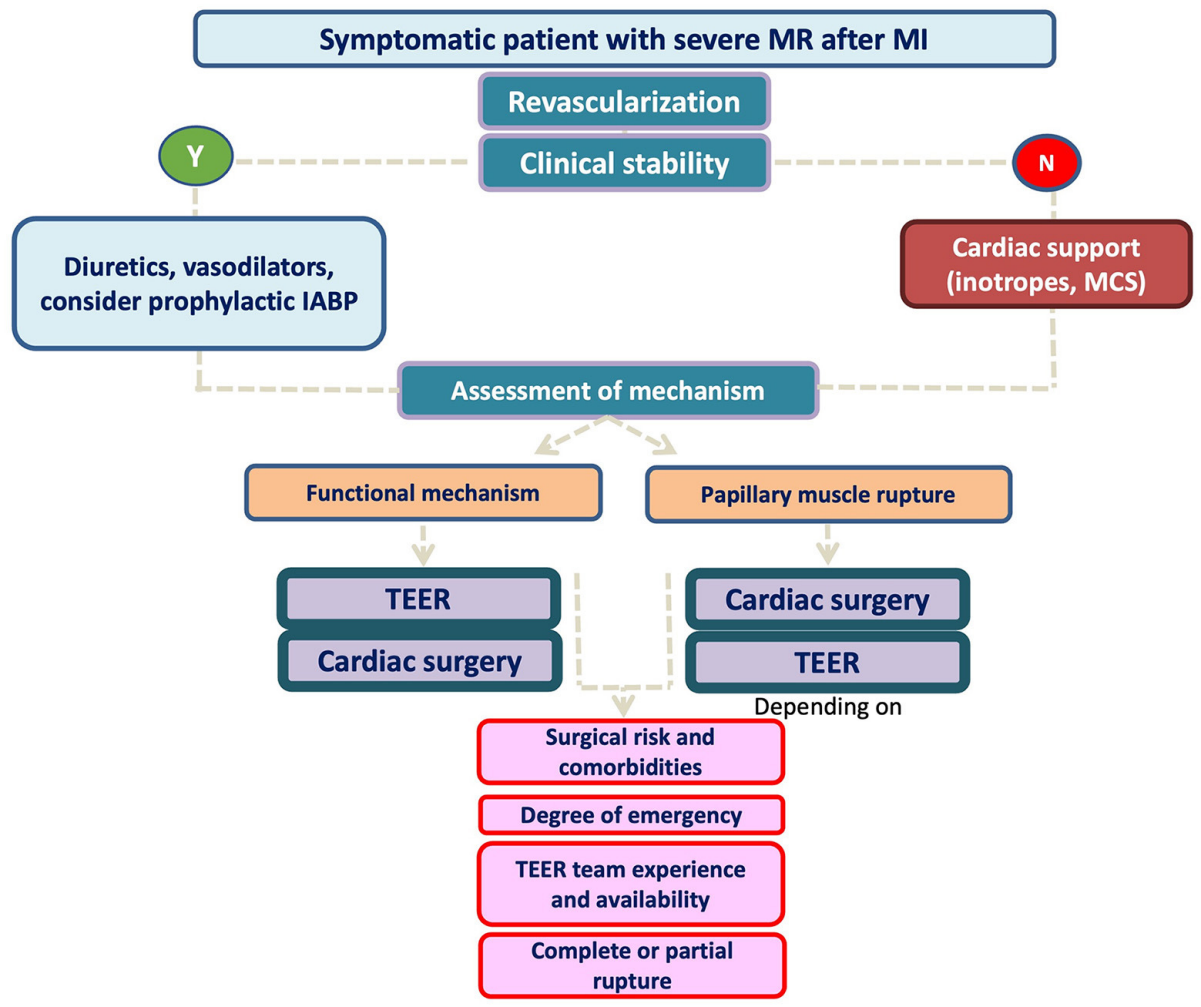
Proposed algorithm for post MI MR management.

A proposed algorithm for MR management including all available information is presented in Figure 5. Patients with subvalvular apparatus rupture should probably be first referred to conventional surgery unless the surgical risk is high and the valve anatomy is suitable for TEER (taking into account team experience). Conversely, patients with more “functional-type” MR seems to perform better with TEER as a first strategy and only patients with suboptimal valve anatomy can be first considered for open-heart surgery, if the risk is acceptable.

## Future developments

As long as this is a novel therapy in the interventional field, there are still multiple unsolved issues. First, the time elapsing from MI or MR to TEER is very long in most of cases. This fact is due to the belief that MR will improve after revascularization in most cases or that patients are too sick to receive treatment. Data shows that the procedure is associated with a high technical success, and this translates in rapid recovery of clinical condition and therefore it should not be delayed. Likewise, it is likely that with earlier treatment the clinical result could be potentially better. Therefore, if MR is severe, associated with regional LV remodeling and the patient is symptomatic, is unlikely that the valve disease will resolve under medical management and the treatment should not be delayed. In our opinion, in those cases where heart team is prone to percutaneous treatment, the concept of Primary TEER (similar to primary PCI) should be implemented to avoid delays. Second, the role of mechanical cardiac support in patients with hemodynamic instability depends on the moment of development of MR. If MR is present at the time of coronary angiography and revascularization VA ECMO is not advisable since it increases the afterload and this can worsen the pulmonary edema. IABP/Impella and prompt MR correction are more advisable. However, those patients initially in shock and under ECMO can develop MR during follow-up. In those cases, ECMO weaning is advisable to ensure the severity of MR. In cases that this cannot be done, TEER under ECMO is safe and feasible. In such cases, LV may be unloaded with a Impella combination (ECPELLA strategy), with potential benefits to TEER treatment (lower LV dimension, coaptation gap and less severe MR). And third, we only have information with the MitraClip device. In the last years different devices have gained space in the field both from repair, where PASCAL is showing promising results (50, 51), and from replacement (52, 53). The role of PASCAL should be similar to MitraClip but the role of replacement still needs further development.

## Conclusion

TEER with MitraClip has shown to be an efficacious treatment for early MR after MI, in a selected group of patients. The possibility of a transcatheter correction in this scenario should be present in all algorithms of management of post MI mitral insufficiency, together with surgical option, for the heart team to have all available options of treatment.

## Author contributions

RE-L, JB-A, FC-I, and MT conceived the idea. RE-L, BC-Q, and MB-P drafted the manuscript. RG-F and LP corrected the manuscript and assisted in the figures. MP and AÍ-R gave critical review. All authors gave final approval.

## Conflict of interest

The authors declare that the research was conducted in the absence of any commercial or financial relationships that could be construed as a potential conflict of interest.

## Publisher's note

All claims expressed in this article are solely those of the authors and do not necessarily represent those of their affiliated organizations, or those of the publisher, the editors and the reviewers. Any product that may be evaluated in this article, or claim that may be made by its manufacturer, is not guaranteed or endorsed by the publisher.
